# Single-Gene Deletions Contributing to Loss of Heterozygosity in *Saccharomyces cerevisiae*: Genome-Wide Screens and Reproducibility

**DOI:** 10.1534/g3.119.400429

**Published:** 2019-07-03

**Authors:** Kellyn M. Hoffert, Erin D. Strome

**Affiliations:** Department of Biological Sciences, Northern Kentucky University, Highland Heights, Kentucky, 41099

**Keywords:** *S. cerevisiae*, loss of heterozygosity, MAT, *MET15*, screen comparison

## Abstract

Loss of heterozygosity (LOH) is a phenomenon commonly observed in cancers; the loss of chromosomal regions can be both causal and indicative of underlying genome instability. Yeast has long been used as a model organism to study genetic mechanisms difficult to study in mammalian cells. Studying gene deletions leading to increased LOH in yeast aids our understanding of the processes involved, and guides exploration into the etiology of LOH in cancers. Yet, before in-depth mechanistic studies can occur, candidate genes of interest must be identified. Utilizing the heterozygous *Saccharomyces cerevisiae* deletion collection (≈ 6500 strains), 217 genes whose disruption leads to increased LOH events at the endogenously heterozygous mating type locus were identified. Our investigation to refine this list of genes to candidates with the most definite impact on LOH includes: secondary testing for LOH impact at an additional locus, gene ontology analysis to determine common gene characteristics, and positional gene enrichment studies to identify chromosomal regions important in LOH events. Further, we conducted extensive comparisons of our data to screens with similar, but distinct methodologies, to further distinguish genes that are more likely to be true contributors to instability due to their reproducibility, and not just identified due to the stochastic nature of LOH. Finally, we selected nine candidate genes and quantitatively measured their impact on LOH as a benchmark for the impact of genes identified in our study. Our data add to the existing body of work and strengthen the evidence of single-gene knockdowns contributing to genome instability.

Genome instability underlies a multitude of changes observed during tumorigenesis. The accumulation of mutations, both as drivers and as a result of tumor formation, give important insight into cancer progression (Loeb *et al.* 2003). For most genes conferring a protective effect against tumorigenesis, loss of function alterations need to occur in both alleles before the development of cancer phenotypes (Kacser and Burns 1981; Knudson 1993). However, some genes have been discovered to impart haploinsufficient effects whereby a mutation event in only one allele leads to an abnormal cellular phenotype (Veitia 2002; Trotman *et al.* 2003; Alimonti *et al.* 2010; Berger *et al.* 2011). In most cancers, the accumulation of mutations leading to tumorigenesis does not happen only at the single nucleotide level, but with large chromosomal gains or losses (Lengauer *et al.* 1998; Loeb *et al.* 2003; Giam and Rancati 2015; Gao *et al.* 2016). Loss of heterozygosity (LOH) events are one such type of genome instability implicated in tumorigenesis and can arise from a myriad of underlying mechanisms, Figure 1, (Knudson 1971; Vladusic *et al.* 2010; Choi *et al.* 2018a) for additional review: (Levine 1993; Thiagalingam *et al.* 2002; Payne and Kemp 2005).

**Figure 1:**
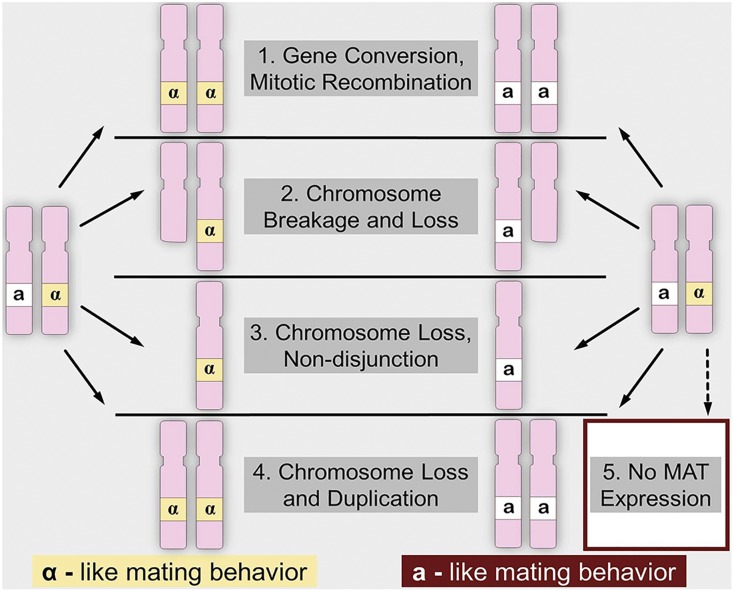
Representative LOH events impacting the MAT locus that can result in diploids exhibiting haploid mating behavior. Due to the co-repressible nature of the MAT locus, both *MATa* and *MATα* alleles must be present and active in order to suppress the production of mating pheromones and receptors, leading to the non-mating diploid phenotype.

Identifying driver mutations responsible for LOH events has proven a difficult task in mammalian cells; by the time tumors are large enough to be detected and examined, tens if not hundreds to thousands of mutations have occurred. Utilizing *Saccharomyces cerevisiae* for studying genomic instability mechanisms, including LOH, has long been an invaluable resource in furthering our understanding of molecular underpinnings (for review see (Mager and Winderickx 2005; Botstein and Fink 2011)). Much of the knowledge gained through yeast can then be applied to higher eukaryotic organisms.

With the creation of the *S. cerevisiae* gene knockout collections, large scale surveys for genes implicated in a particular phenotype can be systematically conducted (for review (Giaever and Nislow 2014)). The 6,477 strains included in the heterozygous deletion collection (hereafter referred to as SCDChet) target both essential and non-essential genes. In this screening, we use SCDChet to systematically screen for genes with a haploinsufficient effect resulting in increased LOH events. We first utilize the mating type (MAT) locus, on chromosome III, which is endogenously heterozygous, *MATa*/*MATα*, in diploid yeast cells. The non-mating diploid phenotype results from a co-repressive mechanism driven by the *MATa* and *MATα* alleles (Duntze *et al.* 1970; Herskowitz 1995; Schmidlin *et al.* 2007; Haber 2012). When an LOH event occurs at this locus, the co-repressible mechanism is no longer active to prevent mating, and the diploid cell mates as if it were haploid. As a secondary assay to confirm increases in LOH events, mutants identified as top-hits for LOH at the MAT locus were tested for increases in LOH at the separate *MET15* locus on chromosome XII. The SCDChet is heterozygous at *MET15* (*met15Δ/MET15*) and a color-identifying sectoring assay allows LOH at this locus to be readily measured (Cost and Boeke 1996).

The MAT and *MET15* loci have been exploited as markers in other LOH screens interested in identifying heterozygous and/or homozygous knockouts that increase genome instability (Yuen *et al.* 2007; Andersen *et al.* 2008; Choy *et al.* 2013). Our goals were therefore twofold: first to identify genes with haploinsufficiency impacts on genome stability, and second, in performing a variety of follow-up analyses, to identify those top-hits with the most potential to be cancer susceptibility genes. These follow-up analyses include gene ontology characterizations, positional gene enrichment identification, and multiple comparisons to other screens interested in identifying genes with impacts on genome stability, to determine the enrichment and reproducibility of gene identification. Thereby not only giving insight into candidate cancer susceptibility genes that have a haploinsufficient impact, but also deepening our understanding of the differences in results due to variations in screening set-ups and the stochastic nature of LOH events. By extensively documenting the methodology used, as well as comparing datasets across multiple screens, we aim to sort through much of the noise of screen results and avoid the far too common crisis of reproducibility (Yamada and Hall 2015; Drucker 2016).

## Materials and Methods

### Creation of mating tester haploids

One *MATa* haploid *(MATa*: *his3-∆200*, *trp1-∆1*, CF:[*ura3*: TRP1 SUP11 CEN4 D8B], *leu2-3*, *lys2-801*, *ura3-52*, *ade2-101)* and one *MATα* haploid (*MATα*: *his3-∆200*, *trp1-∆1*, CF:[*ura3*: TRP1 SUP11 CEN4 D8B], *leu2-3*, *ura3-52*, *ade2-101)* were transformed using lithium acetate transformation with a HIS3MX cassette, carrying the *Schizosaccharomyces pombe HIS5* gene, to replace the wildtype *CAN1* locus. This resulted in the production of haploid strains EDS585*a* (*MATa*: *his3-∆200*, *trp1-∆1*, CF:[*ura3*: TRP1 SUP11 CEN4 D8B], *leu2-3*, *lys2-801*, *ura3-52*, *ade2-101*, *can1*::*HIS5)* and EDS588*α (MATα*: *his3-∆200*, *trp1-∆1*, CF:[*ura3*: TRP1 SUP11 CEN4 D8B], *leu2-3*, LYS2, *ura3-52*, *ade2-101*, *can1*::*HIS5)* hereafter referred to as 585*a* and 588*α*.

### Heterozygous deletion collection (SCDChet) screen for LOH at the MAT locus

**Figure 2:**
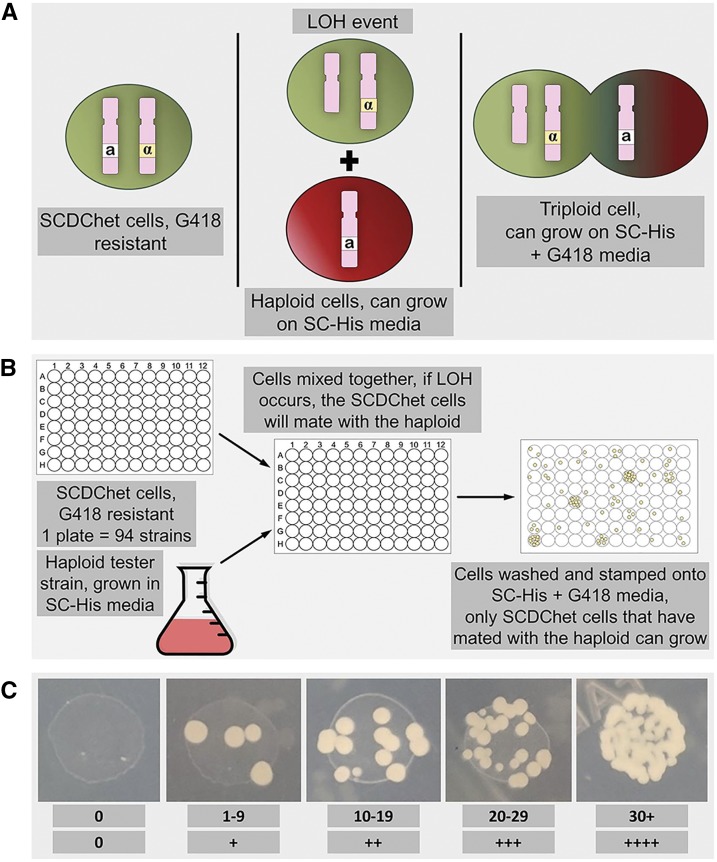
Theory and set-up of LOH screen at the MAT locus. A) Example of a possible triploid cell formation mechanism that would allow for growth under double selection conditions. The LOH event could occur through a variety of mechanisms, all leading to the ability of the diploid cell to mate. B) Model of the MAT locus LOH screening methodology. C) The scoring system of colony counts utilized in the screening. Strains demonstrating a minimum score of “+++” in all four replicates for mating with a particular haploid mating type were included as a top-hit.

The heterozygous deletion collection (6,477 strains) was purchased from Open Biosystems (YSC1055). Three initial copies of the collection were made (200 μL YPD + G418 (0.2 mg/mL)); 10 μL of cells were transferred to a 96-well plate, grown overnight at 30°, 60 µL of 67% glycerol was added and plates were kept were frozen at -80°. Haploid strains *585a* and *588α* were struck from freeze-down stocks and grown for 3 days. One colony from each strain was inoculated individually into 100 mL SC-His and grown overnight on a shaker. On the same day, deletion collection plates were thawed and new copies were made of each plate grown in 200 μL YPD + G418 (0.2 mg/mL) with 20 μL starting inoculum of deletion collection cells. These copies were incubated overnight at 30° to allow LOH events to occur. The deletion collection cells were mixed with either *585a* or *588α* haploid cells (200 μL YPD, 10 μL SCDChet cells, 10 μL haploid cells) and grown for 24 hr at 30° to allow mating events to occur. The cells were then spun down at 2,500 rpm for 4 min, the supernatant was removed, and the cells were washed with 200 μL ddH_2_O. The cells were again spun down at 2,500 rpm, the supernatant was removed, and the cells were resuspended in 200 μL ddH_2_O. Resuspended cells were pinned to SC-His + G418 (0.4 mg/mL) in duplicate and incubated for 48 hr at 30° before colony counts were taken. A scoring system was utilized to record colony count ranges. Pinned spots with no colonies were recorded as “0,” 1-9 colonies were scored as “+,” 10-19 as “++,” 20-29 as “+++,” and any colony counts above 30 were scored as “++++” (Figure 2C). Two complete trials for each haploid mater were conducted, utilizing different copies of the SCDChet collection for each trial.

The methodology used in this screening was verified through a series of control experiments. *585a* and *588α* were put through the screening protocol as described above, but without SCDChet cells introduced to the samples. Haploid tester strains were grown in duplicate and 20 µL samples were placed into 96-well plates. None of the 192 wells per haploid pinned onto SC-His + G418 (0.4 mg/mL) contained colonies, indicating that false positives of the haploid testers growing alone on double selection plates are unlikely. To screen for false positives originating from SCDChet cells, five SCDChet plates were selected at random, and put through the screening protocol above without the addition of a haploid tester. Of the 413 unique strains pinned, 23 samples contained colonies. Fourteen of these strains were identified as producing lawn growth, not single colonies, interpreted as their starting with a genotype conferring an ability to grow on SC-His + G418 (0.4 mg/mL) without needing to mate with *585a* or *588α*. (When assayed again in the full screen, these 14 wells continued to show lawn growth when incubated with either the *585a* or *588α* strains.) Therefore, these wells were excluded as top-hits, as well as any other wells that showed lawn growth, after incubation with both *585a* and *588α*. The wells identified with this characteristic can be found in Table S1. Of the remaining nine wells that contained colonies, not one contained a background level of growth to score above a “++” on the scale established. This indicates a low level of false positives – approximately 2% - if classifying any amount of growth as a positive hit. This then informed our decision on a threshold for ‘top-hits’ from our screen (as discussed later), ensuring these types of events are not enough to warrant categorization as a ‘top-hit’ to mark a gene as one of interest. *585a* and *588α* were also mated with control haploids containing KANMX cassettes to determine false negative rates. Two different control *MATa* and two different control *MATα* strains, with KANMX cassettes inserted at different locations in the genome, were utilized. All conditions were kept the same as the screening described above, but instead of inoculating SCDChet cells, the KANMX-containing haploid controls were inoculated into 100mL YPD + G418 (0.2mg/mL). Opposite mating types were paired. All pairings, 32 samples in total, resulted in uncountable lawn growth or colony counts greater than thirty (++++ score). This verifies strain mating occurs in the incubation time allotted, scoring at the top level of growth, and giving no indication of false negatives.

### Secondary screen for LOH at MET15

Methodology was adapted from previous screenings (Ono *et al.* 1991; Cost and Boeke 1996; Andersen *et al.* 2008); briefly, the 217 top-hits identified in the MAT locus LOH assay were additionally evaluated to determine if LOH was also increased at a secondary locus. When cells lacking functional *MET15* are placed on plates containing lead (Pb^2+^), the excess sulfur precipitates as dark-colored lead (II) sulfide (PbS) (Cost and Boeke 1996). Cells must contain at least one functional copy of *MET15* to remain white in appearance; cells that have undergone LOH at *MET15* and no longer maintain a functional copy will appear as a black/brown sector. Two microliters of each SCDChet top-hit were inoculated into 200µL YPD and incubated for three days at 30° to allow LOH events to occur. Cells were then pinned to plates in triplicate containing 0.7mg/mL lead nitrate (for full plate recipe see Guide to Yeast Genetics and Molecular Cell Biology, Elsevier 2002), and allowed to grow at 30° for four days. Plates were then placed at 4° for 24 hr to aid in color development before sectors were counted. As with other published methodologies (Ono *et al.* 1991; Cost and Boeke 1996; Andersen *et al.* 2008), pinned samples grew as patches, not individual colonies, and sectors are counted as the total number of dark growth regions within the larger patch. The screen was run twice (two biological replicates); strains were scored as positive for LOH at *MET15* if an average of more than 13 sectors appeared in each replicate across the 6 replicates when analyzed at 24x magnification. Eight mutants (*KIN3*, *MET17*, *STH1*, *MVD1*, *HRB1*, *BSC5*, *VAM6*, and *YKR073C*) were unable to be analyzed for sectoring due to presenting as entirely brown colonies from the initial pinning to the lead-containing plate.

The methodology used in this screening was verified through a series of control experiments. To determine the baseline rate for LOH at *MET15* for the SCDChet collection, we utilized two biological replicates of a plate chosen from the SCDChet collection that contains no strains identified as increasing LOH at MAT in our primary screening assay. In an attempt to ensure that LOH events that happened earlier, and thus resulted in a larger sector portion, were not unduly overrepresented in our results due to increased visibility, we utilized a dissecting microscope for analysis of sectors. Sectors were counted at 24x magnification and averaged across all 94 strains on the plate for 3 technical replicates. Analysis at 24x magnification revealed a high level of sectoring in these strains with the average at 11 sectors. Previous screens utilizing the *MET15* locus and analyzing sector appearance did so without magnification and set their threshold for classification as a hit at 2 sectors (Andersen *et al.* 2008). We therefore set our top-hits threshold level at +2 over the average seen in this control set of analyzed strains. The top-hit threshold was therefore set at having 13 or more sectors as indicating an increase in LOH over the baseline.

It has been previously documented (Bae *et al.* 2017) that the SCDChet strain that contains the knockout for *YLR303W/MET17* (alias of *MET15*) is genotyped as *met15∆*::*kanMX/met15∆*. Due to its lack of functioning MET15, all cells of this strain plated on Pb^2+^-containing plates should appear entirely black/brown. This phenotype was confirmed in all replicates (two biological, three technical) where this strain was subjected to the screening protocol. This strain serves as a positive control for this secondary screening.

### Fluctuation analysis benchmarking of top candidate gene deletions

The parental strain used to create SCDChet was ordered from Open Biosystems (BY4743). This strain underwent lithium acetate transformation with a *can1*::*HIS3* cassette in order to render the CAN1 locus heterozygous. Mutant strains containing the heterozygous knockouts of *SSA1*, *CCT7*, *MRC1*, *RSM7*, *HSL7*, *SSE2*, *PUG1*, *YOR364W*, and *MEC1* were grown from SCDChet and subjected to the same *can1*::*HIS3* switch out via lithium acetate transformation. Strains were then struck for individual colonies and grown at 30° until they reached ∼3mm, allowing LOH events to occur. Twenty-four individual colonies per strain were then resuspended in water, assessed for optical density, and the 15 colonies with the most similar size, as read by absorbance at 562nm greater than 0.5, were used for analysis. The rate of LOH events resulting in a change to the CAN locus were determined by plating dilutions on non-selective (YPD for population size) and SC-Arg^-^ plus canavanine (60 μg/mL) plates (for LOH events). Plates were grown for 3-5 days at 30°, followed by colony counting. Fluctuation analysis for LOH rate with 95% confidence intervals (CI) were calculated utilizing the R advanced calculation package Salvador (rSalvador) (Zheng 2002, 2008, 2016). The LOH rates and confidence intervals were measured for two biological replicates for each strain.

### Data availability

Strains are available upon request. Table S1. Table of complete MAT locus LOH screen data. Table S2. Top hits identified in MAT locus LOH screen. Table S3. Sector counts of the 217 SCDChet gene deletions analyzed in secondary *MET15* screen. Table S4. GO Slim Mapper results for the 217 identified genes in the MAT locus screen. Table S5. GO Slim Mapper results for the 100 identified genes in the *MET15* secondary screen. Table S6. GO Slim Mapper and GO Term Finder results for the 91 genes identified in two or more related independent studies. Table S7. Positional Gene Enrichment analysis of the 217 MAT Top Hits, 100 MAT + MET15 Top hits, and 91 genes identified in 2+ independent studies. Table S8. Human homologs and their associated cancer types for genes identified by the MAT locus screen. Table S9. Human homologs and their associated cancer types for genes identified by multiple independent studies. Table S10. Positional Gene Enrichment analysis of genes identified by similar independent studies. Figure S1. Visual representation of Positional Gene Enrichment data for enriched region on chromosome II. Supplemental material available at Figshare: https://doi.org/10.25387/g3.8325992.

## Results

### MAT locus screen for genes with haploinsufficiency effects on LOH

To identify genes that, when heterozygously mutated, resulted in increased incidence of LOH at the *MAT* locus, the SCDChet collection was screened. SCDChet cells were grown in 96-well plates and paired separately with *MATa* and *MATα* haploid tester strains. When pinned onto double selection media, only cells that had mated and formed triploids grew (Figure 2A and 2B). The entire collection was screened twice (biological replicates) with technical replicates performed each time. Furthermore, this was done with both *MATa* and *MATα* haploid maters resulting in 8 data points for each SCDChet strain. Each trial stamped on SC-His + G418 plates was counted independently for growth and assigned a score of 0, +, ++, +++, or ++++ (Figure 2C). The scores of all four replicates were summed for an SCDChet strain with given haploid pairing; any combination of “+” scores adding to 12 or higher were further analyzed to be considered as a ‘top-hit’. For comparison, across the whole SCDChet collection, the average amount of growth seen on the double-selection plates scored as a single “+” for any one spot, and a summed plus score of three across the four replicates. Further, any pairing that resulted in a score of twelve, but contained a replicate that scored “++” or below was removed from top-hit consideration due to the possibility of it being a false positive. Strains that were annotated in the SCDChet database provided by Open Biosystems as having a phenotype related to mating were excluded from consideration as a top-hit, as their ability to mate did not represent an LOH event, but an inherent characteristic of that particular mutant. This screening mechanism resulted in 217 heterozygous gene mutations, approximately 3.4% of the genome, being scored as top-hits (Table S2). For listing of all scores of all strains from this study, see Table S1.

### Secondary screen for haploinsufficiency effects on LOH at an alternate Locus (MET15)

To further understand the extent of the impact of these 217 top-hit heterozygous gene mutations on LOH events, these strains were screened for their LOH impacts at an alternate secondary locus; the *MET15* gene on chromosome XII. Each identified top-hit SCDChet strain was grown up in a 96-well plate for three days, pinned to lead-containing plates, then grown up into a patch over four days until color developed. When pinned onto plates containing Pb^2+^, cells within a patch that have undergone an LOH event will appear as black/brown sectors. All 217 top-hit SCDChet strains were tested twice (biological replicates) and stamped onto lead-containing plates in triplicate each time (technical replicates), resulting in 6 sector counts for each mutant strain. One hundred of our initial 217 top-hit heterozygous gene deletions induced increased LOH events at the secondary *MET15* locus. This indicates that ∼48% of our intiallially identified heterozygous gene mutaitons have large reproducible effects on LOH at multiple loci, representing 1.5% of the genome. Eight of the initial 217 strains were unable to have sectors counted for LOH at *MET15* due to presenting a brown phenotype throughout the entire patch. See Table S3 for *MET15* sectoring data for each tested strain.

### Multiple screen comparison to identify reproduced results from independent studies assessing LOH and/or haploinsufficiency induced genome instability

**Figure 3:**
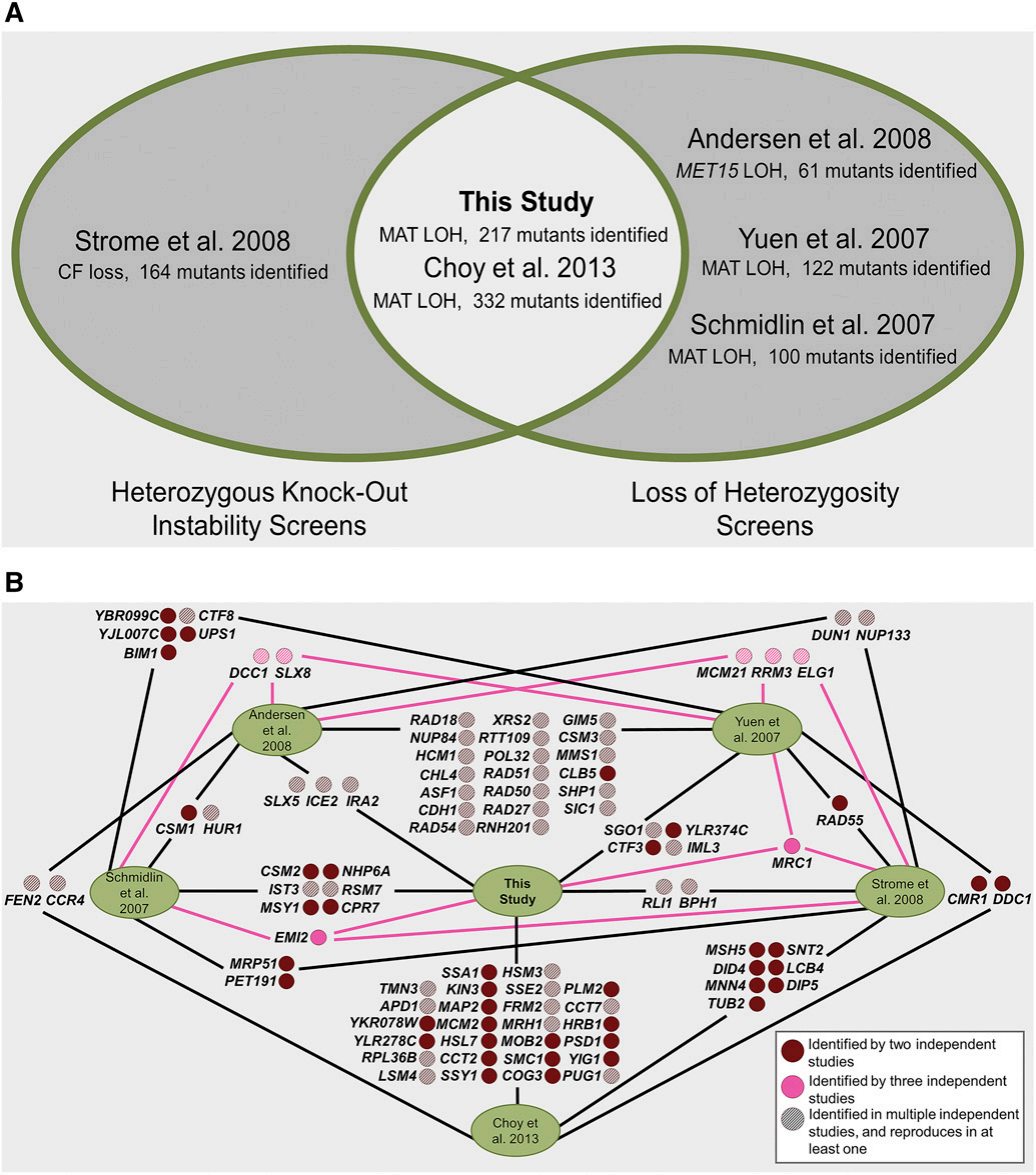
Multiple independent screen selection and gene lists comparison. A) Data sets were compared across screens that either assayed heterozygous deletions, assayed for LOH events, or both. Screens looking at haploinsufficiency and its connection to genome instability, or utilizing homozygous knockouts to understand LOH mechanisms were chosen as they provide the most relevant data sets for comparison. B) Gene lists from the six screens selected for comparison were mapped for their overlapping top-hits. Gene deletions identified in two independent studies are shown in burgundy, whereas genes appearing as hits in three independent studies are shown in pink. If a particular gene deletion reproduced in multiple screens within the same publication, the solid color was changed to a striped pattern. Only screens performed in a diploid system were considered when determining the number of screens a gene reproduced in.

To further expand our analysis of the reproducibility of gene identification, we compared our results to previously reported screens that asked similar questions about genes contributing to genome instability. Screens were selected for comparison based on a) utilizing heterozygous knockouts to screen for genome instability, b) screening homozygous knockouts specifically for *LOH*, or c) a combination of the two (for a visual representation of screen selection, see Figure 3A).

#### Choy et al:

The greatest number of overlapping hits, 26, were seen when our results from the MAT locus screen were compared to a screen that most mirrored our own, having utilized the SCDChet to screen for LOH at the MAT locus, Figure 3B, (Choy *et al.* 2013). This overlap shows statistical significance at the level of *P* = 3.995 × 10^−5^ when a hypergeometric probability was calculated using a normal approximation, Table 1. (Ten of the overlapping genes, *APD1*, *SSE2*, *HSM3*, *FRM2*, *MRH1*, *LSM4*, *TMN3*, *PUG1*, *RPL36B*, and *CCT7*, also reproduced in our secondary screen for increasing LOH at *MET15*). This screen arrayed four samples from each well of the SCDChet onto solid YPD media with either a *MATa* or *MATα* haploid tester to allow for mating. The mating results for each pinned position were then summed to result in a score of 0-4 for each. The entire screen was performed in triplicate, strains that had a score ≥ 2 SD above the mean were included as top-hits.

**Table 1:** Multiple Screen Comparison Gene Identification Overlap. A hypergeometric probability was calculated using a normal approximation using the webtool http://nemates.org/MA/progs/overlap_stats.html. The same tool was used to calculate a representation factor. A representation factor is calculated as the number of genes in common between two studies divided by the number of expected genes. The number of expected genes is estimated as the number of genes in the first study times the number of genes in the second study which is then divided by the total number of genes that were screened. Representation factors greater than 1 indicate more overlap than expected, representation factors less than 1 indicate less overlap than expected

Screen	Total Number of Genes Screened	Number of Mutants with Phenotype (“hits”)	Number Overlapping with This Study “hits”	p-value	Representation Factor
This Study	6477	217 (180*)	—	—	—
Choy *et al.* 2013	6477	332	26	0.00003995	2.3
Strome *et al.* 2008	6477	164	4	0.351	0.7
Andersen *et al.* 2008	5134	61	3	0.362	1.4
Yuen *et al.* 2007	5134	122	5	0.427	1.2
Schmidlin *et al.* 2007	5134	100	7	0.060	2.0

* 217 total top-hit genes identified, 180 non-essential top-hit genes were used for comparison with homozygous knockout screens.

#### Strome et al:

Previously an independent screen was conducted using random insertional mutagenesis generated heterozygous knockouts, to identify gene mutations involved in genome instability, specifically chromosome transmission fidelity (CTF) (Strome *et al.* 2008). Heterozygous knockouts containing a chromosome fragment (CF) allowed for visual identification of fragment loss due to insertional mutagenesis induced genome instability (Hieter *et al.* 1985). The SCDChet was not used in this screening. Of the 164 hits identified, 4 of them (2.4% of their 164) overlap with genes we have identified for increasing MAT LOH, representing 1.8% of our dataset, Figure 3B. Two of the identified gene deletions – *RLI1* and *BPH1* – also increased LOH at *MET15*.

#### Andersen et al:

Screens that assayed for LOH events even if not utilizing heterozygous deletions can still provide valuable insight. This study utilized the homozygous deletion collection (SCDChom) and looked at LOH events at three different loci. Their primary screen utilized the intrinsic heterozygosity at the *MET15* locus in SCDChom to measure LOH. To further examine genes of interest identified in their initial screen, they constructed a strain with Many Heterozygous Markers (MHM) on chromosomes III, IV, and XII to understand the extent of the events at various loci. From the initial *MET15* screening in SCDChom, they identified 132 gene deletions that resulted in increased LOH. They were able to successfully recreate 114 of these knockouts in their MHM background, which they screened again for *MET15* loss at elevated rates. Of the 114 MHM knockouts, 61 of them again demonstrated elevated LOH at *MET15*. These 61 knockout strains were then examined further for the extent of their LOH activity at two additional loci, *SAM2* and MAT. Additional assays identified 26 of these genes with effects increasing LOH events across three independent loci. In comparing all genes identified as increasing LOH from their screening data, we observe three overlaps with our gene list, *SLX5*, *IRA2* and *ICE2*, Figure 3B. A representation factor (Rf) calculated for this overlap indicates a value greater than 1, (Rf = 1.4), indicating more overlap than expected, although not at a p-value <0.05, Table 1. Our secondary *MET15* screen also identified *SLX5* and *ICE2* knockdowns as contributing to increased *MET15* LOH. These three genes represent a 4.9% overlap in their dataset and 1.7% of our data (when corrected to remove essential genes not identifiable in their screening method).

#### Yuen et al:

Another screen looking at LOH events due to homozygous mutations once again utilized SCDChom but examined the presence of the bi-mater (BiM) phenotype (among other genome instability assays) (Yuen *et al.* 2007). The BiM assay measures LOH events at the MAT locus that allow for mating with haploid testers of both mating types. When comparing top-hits between our screens, 5 genes overlap as having elevated levels of LOH, Figure 3B. A representation factor (Rf) calculated for this overlap indicates a value greater than 1, (Rf = 1.2), indicating more overlap than expected, although not at a p-value <0.05, Table 1. This represents 2.8% of top-hit data from our screen (corrected to remove essential genes not identifiable with the SCDChom), and 4.1% of overlap from their list of identified genes that lead to a BiM phenotype. Two genes identified in both screens – *IML3* and *SGO1* – reproduced in our secondary *MET15* screening.

#### Schmidlin et al:

A third screen utilizing SCDChom again analyzed mating capabilities with haploid testers to examine LOH (Schmidlin *et al.* 2007). Mating pairs of SCDChom cells and a haploid tester were pinned to plates of minimal medium that allowed for triploid selection; growth of four or more colonies in a sample was considered a positive hit. One hundred homozygous gene deletions were identified in this initial screen, and seven of those overlap with top-hits found in our screening, Figure 3B. A representation factor (Rf) calculated for this overlap indicates a value greater than 1, (Rf = 2.0), indicating more overlap than expected, although at a p-value = 0.06, Table 1. Only including our non-essential hits, this represents 3.9% of our dataset, and 7% of the strains initially identified in their screening. Eighty-nine homozygous deletions were then remade by mating the corresponding deletion collection haploid strains to make new homozygous diploids in the same background. In assays to confirm the mating phenotype, six reconfirmed. One of the six genes they identified overlapped with one of our hits – *IST3. IST3*, as well as *RSM7* (one of the genes identified in their initial screen), were identified in our secondary screen as increasing the rate of LOH at *MET15*.

While the stochastic nature of LOH events, as well as the differences in the instability phenotypes being assayed, contribute to the limited overlap between screens, genes that consistently appear as top-hits in screens interested in similar instability mechanisms provide interesting avenues for further investigation. Figure 3B shows the results of the comparison of each of these individual screens to each other and the 91 genes found minimally in two independent publications, seven of which were identified in three studies. Furthermore in the current climate of questions surrounding reproducibility we are pleased to see a significant level of overlap between our screen results and the Choy *et al.* screening for MAT locus LOH with SCDChet strains; approximately 11.9% of our top-hit genes were identified in their results and this represents 7.8% of their dataset.

### Gene ontology analysis for identified enrichment categories

To achieve a primary understanding of the functions carried out by members of our first list of top-hits, we analyzed this 217-member *MAT* locus LOH gene list using two Gene Ontology (GO) tools: *Saccharomyces* Genome Database (SGD) GO Slim Mapper and GO Term Finder. GO Slim Mapper provides an overview of broader parent terms that a gene can be mapped to, selected by SGD curators, and does not automatically generate enrichment p-values based on its grouping of genes into categories. Fisher’s exact test, with the Benjamini-Hochberg correction for false discovery rates (FDR) (Q = 0.05) were selected and applied based on their documented use for ontology analysis (Benjamini and Hochberg 1995; Sabatti *et al.* 2003; Rivals *et al.* 2007). Significant results from SGD GO Slim Mapper (p-value < 0.05, and *P* < Benjamini-Hochburg critical value) are summarized in Table 2A. Four ontology varieties of SGD GO Slim Mapper were utilized: Cellular Component, Molecular Function, Biological Process, and Macromolecular Complex. Alternatively, SGD GO Term Finder selects the most granular term for each gene within a query, providing as detailed of an analysis as currently available based on the literature (Christie *et al.* 2009). GO Term Finder uses a binomial distribution to calculate p-values corrected for multiple comparison analysis, and there are three varieties of annotations: Cellular Component, Molecular Function, and Biological Process. Many of the categories found in GO Slim Mapper Macromolecular Complex are absorbed into the more specific GO Term Finder Cellular Component, but GO Term Finder and GO Slim Mapper analysis were kept separate due to the nature of the algorithms and the statistical analysis of each dataset.

**Table 2:** Go Slim Mapper and Go Term Finder Results. A. 217 MAT Top Hits List Significantly enriched gene ontology categories identified with SGD Slim Mapper and SGD Term Finder tools are shown. SGD Term Finder reported multiple comparison corrected p-values < 0.05 are shown. P-values from SGD Slim Mapper were corrected with the Benjamini-Hochberg critical value. For SGD Slim Mapper, all significantly enriched categories with a uncorrected p-value < their Benjamini-Hochberg critical value (Q = 0.05) are shown. Gene Ontology Identification numbers (GOID) and names of the genes that represent the enrichment are included

GO Categories	GO Method	GO Term	p-value	Benjamini-Hochburg critical value (Q = 0.05)	Genes in term
Cellular Component	SGD Slim Mapper	—	—	—	—
	SGD Term Finder	Chaperonin-containing T-complex (GOID: 5832)	0.04889	—	SSA1, CCT2, CCT8, CCT7
Biological Process	SGD Slim Mapper	Chromosome segregation (GOID: 7059)	0.0004605	0.00049505	KIN3, IML3, MRC1, MPS1, SPC19, SMC1, KIP3, SPO22, STH1, CSM2, HSK3, CTF3, TUB1, NDJ1, SGO1, KIN4, GPN2
	SGD Term Finder	—	—	—	—
Molecular Function	SGD Slim Mapper	Molecular Function Unknown (GOID: 3674)	0.0004997	0.001136	YAL067W-A, YBR096W, VID24, IML3, YBR137W, YBR144C, APD1, UBS1, HSM3, LDB16, MRC1, BPH1, RMD1, QRI7, RGT2, YDL211C, YDR029W, MRH1, SSY1, UBX5, ECM11, YDR509W, EMI2, GRH1, ZRG8, YER087C-A, TMN3, DSE1, YER135C, BCK2, YER181C, PUG1, SNO3, YGL218W, MTC3, SHE10, NNF2, YGR201C, YHI9, MTC6, AIM18, YIL025C, YIL032C, MMF1, YIL060W, SPO22, AIM19, ICE2, CSM2, SYS1, YJL009W, PRY3, PRM10, YJL120W, SPC1, HIT1, AIM24, ILM1, YKL018C-A, TTI1, FAT3, YKR073C, EMC6, PER33, YLR342W-A, YLR374C, CTF3, YML037C, TUB1, YML094C-A, YMR105W-A, YMR119W-A, YMR122C, YMR153C-A, YNL146C-A, YNL146W, VID27, RTC4, BSC5, YOL134C, MED7, YOR072W, SGO1, AIM41, YOR364W, YPL080C, YIG1, OPY2
	SGD Term Finder	—	—	—	—

GO Categories	GO Method	GO Term	p-value	Benjamini-Hochburg critical value (Q = 0.05)	Genes in term
Cellular Component	SGD Slim Mapper	—	—	—	—
	SGD Term Finder	—	—	—	—
Biological Process	SGD Slim Mapper	—	—	—	—
	SGD Term Finder	—	—	—	—
Molecular Function	SGD Slim Mapper	Molecular Function Unknown (GOID: 3674)	0.000233	0.001136	YAL067W-A, YBR096W, IML3, YBR137W, YBR144C, APD1, HSM3, BPH1, RGT2, YDL211C, MRH1, ECM11, YDR509W, GRH1, ZRG8, YER087C-A, TMN3, DSE1, YER135C, BCK2, YER181C, PUG1, SNO3, NNF2, MMF1, YIL060W, AIM19, ICE2, SYS1, YJL009W, PRY3, YJL120W, SPC1, HIT1, AIM24, YKL018C-A, TUB1, YML094C-A, YMR105W-A, YMR122C, VID27, RTC4, MED7, YOR072W, SGO1, YOR364W, OPY2
	SGD Term Finder	—	—	—	—

The SGD Slim Mapper Gene Ontology tool reported two significantly enriched groups after FDR correction. For the Molecular Function ontology, *Molecular Function Unknown* (p_adj_ = 4.997x10^−4^) was identified as enriched with 88 genes from the 217 top-hits list lacking specific information on their molecular function. Within the Biological Process ontology, *Chromosome Segregation* (p_adj_ = 4.605x10^−4^) was found to be overrepresented with 17 genes classified in this group. To determine if a further understanding of the relationships between the top-hits identified in this screen could be found using more explicit ontology terms, GO Term Finder results were analyzed. The only ontology category that was found to be enriched through GO Term Finder was Cellular Component – *Chaperonin-containing T-complex* (CCT-complex) (p_adj_ = 0.0489). Four genes that are part of this complex were identified in our screening, *CCT2*, *CCT7*, and *CCT8*, three core subunits of the complex, as well as *SSA1*, an ATPase that associates with the core subunits. A complete list of GO Slim Mapper annotations, adjusted p-values, and genes annotated to each term can be found in Table S4.

With the goal of identifiying additional ontologies of interest, SGD GO Slim Mapper and GO Term Finder were also applied to the narrowed list of 100 genes identified as increasing LOH events at two loci. *Molecular Function Unknown* (*P* = 2.328x10^−4^) again reproduced as being significantly enriched in this dataset with 47 genes from 100 top-hit list represented, Table 2B. For the list of all ontologies for the 100 gene list and adjusted p-values of representation see Table S5.

To determine if multiple screen comparisons for repetition of gene identification was likely to lead to ontologies worthy of further pursuit, the 91 genes that were identified in at least two of the independent studies previously discussed, were also analyzed with SGD GO Slim Mapper and GO Term Finder to determine category enrichment. For GO Term Finder Biological Process, 82 categories were annotated as significant (*P* < 0.05), the four categories with largest enrichment are *DNA Metabolic Process* (p_adj_ = 4.63x10^−13^), *Cellular Response to DNA Damage Stimulus* (p_adj_ = 9.12x10^−15^), *Cellular Response to Stress* (p_adj_ = 3.75x10^−13^), and *DNA Repair* (p_adj_ = 1.19x10^−12^). GO Slim Mapper identified 14 Biological Process categories (*P* < 0.05), the four with most significant enrichment are *Organelle Fission* (p_adj_ = 4.79x10^−11^), *Cellular Response to DNA Damage Stimulus* (p_adj_ = 3.44x10^−16^), *Mitotic Cell Cycle* (p_adj_ = 1.04x10^−10^), and *DNA Repair* (p_adj_ = 1.30x10^−14^). Thirty-five categories were enriched for GO Term Finder Cellular Component (*P* < 0.05); the four categories with largest enrichment are *Chromosome* (p_adj_ = 3.25x10^−12^), *Chromosomal Part* (p_adj_ = 4.23x10^−12^), *Nuclear Chromosome* (p_adj_ = 6.29x10^−8^), and *Nucleus (*p_adj_ = 2.41x10^−8^). GO Slim Mapper identified two Cellular Component categories (*P* < 0.05), *Chromosome* (p_adj_ = 3.42x10^−9^), and *Nucleus (*p_adj_ = 4.16x10^−9^). *DNA-Dependent ATPase Activity* (p_adj_ = 0.000228), *DNA Binding* (p_adj_ = 0.00224), *G-quadruplex DNA Binding* (p_adj_ = 0.00416) and *Exonuclease Activity* (p_adj_ = 0.0290) were the significant categories identified using GO Term Finder Molecular Function. While GO Slim Mapper also picked up *DNA Binding* (p_adj_ = 7.41x10-^05^) and *ATPase Activity* (p_adj_ = 0.000808) within the Molecular Function ontology. GO Slim Mapper additionaly found 5 enriched groups in the Macromolecular Complex category (*P* < 0.05), *Chromosome*, *Centromeric Region* (p_adj_ = 1.16x10-^06^), *Kinetochore* (p_adj_ = 0.000135), *SUMO-Targeted Ubiquitin Ligase Complex* (p_adj_ = 0.00019), *Condensed Nuclear Chromosome Outer Kinetochore* (p_adj_ = 0.00019) and *Condensed Nuclear Chromosome Kinetochore* (p_adj_ = 0.000205). For a complete list of GO results from the 91 gene top-hit list of overlaps from the multiple screen comparison, see Table S6.

### Analysis of positional gene enrichment for chromosomal regions of interest

**Figure 4:**
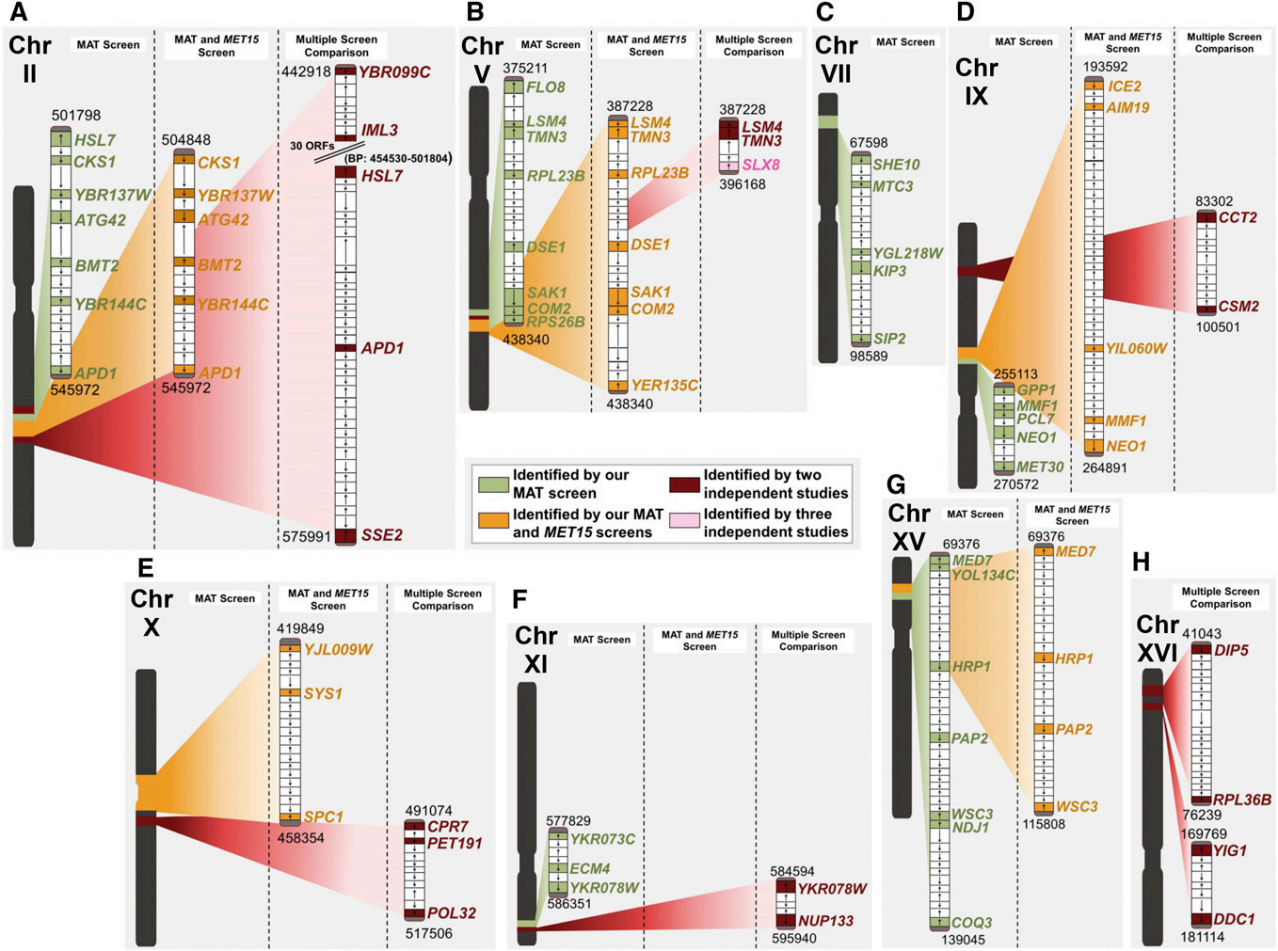
Positional Gene Enrichment (PGE) analysis for enriched chromosome regions. Genes highlighted in green were identified by the MAT screen; genes highlighted in orange were identified by the MAT and MET15 screens; genes highlighted in burgundy were identified in two independent studies; genes highlighted in pink were identified by three independent studies. The key denoting color labels remains the same throughout all parts of the figure. A) Enriched regions of chromosome II from PGE analysis on MAT locus screen top-hits (p_adj_ = 2.74 × 10^−4^), MAT + *MET15* top-hits (p_adj_ = 9.69 × 10^−6^), and Multiple Screen Comparison top-hits (six screens compared in Fig. 3), (BP region 442918-575991) (p_adj_ = *P* = 0.016). B) Enriched regions of chromosome V from PGE analysis on MAT locus screen top-hits (p_adj_ = 2.31 × 10^−4^), MAT + *MET15* top-hits (p_adj_ = 9.69 × 10^−6^), and Multiple Screen Comparison top-hits (p_adj_ = 2.03x10^−5^). C) Enriched regions of chromosome VII from PGE analysis on MAT locus screen top-hits (p_adj_ = 0.00358). No enriched regions of 3 or more identified genes were enriched on this chromosome when the MAT + *MET15* or Multiple Screen Comparison datasets were analyzed. D) Enriched regions of chromosome IX from PGE analysis on MAT locus screen top-hits (p_adj_ = 4.40 × 10^−4^), MAT + *MET15* top-hits (p_adj_ = 0.00194), and Multiple Screen Comparison top-hits (p_adj_ = 0.0299). E) Enriched regions of chromosome X from PGE analysis on MAT locus screen top-hits (p_adj_ = 0.00901) and Multiple Screen Comparison top-hits (p_adj_ = 4.18x10^−4^). No regions containing three or more identified genes were enriched when our MAT locus dataset was run. F) Enriched region of chromosome XI from PGE analysis on MAT locus screen top-hits (p_adj_ = 0.00751) and the Multiple Screen Comparison top-hits (p_adj_ = 0.013). G) Enriched region of chromosome XV from PGE analysis on MAT locus screen top-hits (p_adj_ = 0.00288), and MAT + *MET15* top-hits (p_adj_ = 0.00332). No enriched regions containing more than three identified genes are found on chromosome XV for the Multiple Screen Comparison analysis. H) Enriched region of chromosome XVI from PGE analysis on Multiple Screen Comparison dataset (p_adj_ = 0.046).

To investigate if our screens identified any enriched chromosomal regions, which might be indicative of neighborhoods in the genome that contribute to LOH events, we mapped the location of each gene identified as a top-hit using Positional Gene Enrichment analysis (PGE) (De Preter *et al.* 2008). Genes, which when mutated, identified as top-hits causing increased LOH were dispersed throughout all 16 chromosomes. However, utilizing PGE to assess our different top-hit gene lists allowed us to identify locations with significant enrichment. Analysis of our MAT 217 gene top-hits list identified 41 total locations with significant enrichment, further refinement revealed seven clusters of genes, located on six different chromosomes, with three or more ORFs constituting the enrichment and with multiple comparison adjusted p-values < 0.01 (Figure 4). When we analyzed the narrowed list of 100 mutants that increase LOH at both assayed loci (hereafter referenced as the MAT + *MET15* dataset), 27 total locations were identified significant enrichment, further refinement revealed nine clusters of genes, located on six different chromosomes, with three or more ORFs constituting the enrichment and with multiple comparison adjusted p-values < 0.01. Analysis of our multiple screen comparison 91 gene top-hits list identified 22 total locations with significant enrichment, further refinement revealed two clusters of genes, located on two different chromosomes, with three or more ORFs constituting the enrichment and with multiple comparison adjusted p-values < 0.01. For a list of all enriched chromosomal locations, see Table S7.

#### Chromosome II:

A large region of significance was identified comprised of 20 ORFs found on the right arm of chromosome II (Base Pair (BP) region 501798-545972) (p_adj_ = 2.75 × 10^−4^), when the 217 MAT locus LOH top-hits list was evaluated, as shown in Figure 4A. Seven of the ORFs found in this region were identified by our MAT locus LOH screen – *HSL7*, *CKS1*, *YBR137W*, *ATG42*, *BMT2*, *YBR144C*, and *APD1*. When the MAT + *MET15* data set was subjected to PGE analysis, a more defined region of chromosome II (a subsection of the region mentioned above) was found to be more significantly enriched (BP region 504848-545972) (p_adj_ = 9.69 × 10^−6^) Figure 4A. Six of the seven previously mentioned gene deletions in this region, again appear in a now refined territory comprised of 18 ORFs. We next refined our PGE investigation to all top-hits found in our multiple screen analysis, aiming to better identify chromosomal regions important in LOH events. The region of chromosome II identified above is expanded (BP region 442918-575991) (p_adj_ = 0.016) and contains 5 ORF hits (*YBR099C*, *IML3*, *HSL7*, *APD1*, and *SSE2*) in a 79 ORF region.

#### Chromosome V:

When the 217 top-hits from the initial screen of increasing LOH at the MAT locus were analyzed, a 24-gene region containing eight top-hit ORFs was found to be enriched on chromosome V (BP region 375211-424307) (p_adj_ = 2.31 x10^−4^). These top-hit genes are *FLO8*, *LSM4*, *TMN3*, *RPL23B*, *DSE1*, *SAK1*, *COM2*, and *RPS26B* Figure 4B. The genes *SAK1*, *COM2*, and *RPS26B* are immediately adjacent to one another and are identified as a further enriched cluster with a p-value of 9.57 × 10^−4^. After narrowing our MAT LOH top-hits list to 100 genes that increase LOH at both MAT and *MET15*, one larger region (BP 387228-560360) with 11 hits across a 100-ORF span (p_adj_ = 9.69x10^−6^) and two further enriched subsections of this region (BP region 387228-438340) and (BP region 387228-397649) were identified. The downstream-shifted 30-gene region of chromosome V identified as enriched (BP region 387,228-438,340) (p_adj_ = 9.69 × 10^−6^) is comprised of seven top-hits (*LSM4*, *TMN3*, *RPL23B*, *DSE1*, *SAK1*, *COM2*, and *YER135C*) in the region of 30 genes, while the smaller region is comprised of three ORFs in a six ORF span (p_adj_ = 2.57x10^−4^) Figure 4B. Running the PGE analysis with the overlapping 91 genes from the six compared screens presented a further narrowed enriched region (BP region 387228-396168) (p_adj_ = 2.03x10^−5^), with 3 repeated ORFs (*LSM4*, *TMN3*, and *SLX8*) in a 5 ORF region (Figure 4B). Notably, *LSM4* and *TMN3* were identified by two independent studies (this study and Choy *et al.*), and *SLX8* was identified by three independent studies (Schmidlin *et al.*, Andersen *et al.*, and Yuen *et al.*).

#### Chromosome VII:

An enriched region of significance was identified comprised of 23 ORFs on chromosome VII (BP region 67598-98589) (p_adj_ = 3.57 × 10^−3^), when the 217 MAT locus LOH top-hits list was evaluated, as shown in Figure 4C. Five of the ORFs (*SHE10*, *MTC3*, *YGL218W*, *KIP3*, and *SIP2*) found in this region were identified by our MAT locus LOH screen. When the narrowed list of 100 mutants that increase LOH at both MAT + *MET15* were subjected to PGE analysis this region is no longer identified as being enriched, and further does not recur in PGE evaluation of the 91 overlap list from the multiple screen comparison.

#### Chromosome IX:

An enriched region was identified (BP region 255113 – 270572) when the 217 MAT locus LOH top-hits list was evaluated. This region contains five ORFs (*GPP1*, *MMF1*, *PCL7*, *NEO1*, and *MET30*) identified in a 10 ORF region (p_adj_ = 4.40 × 10^−4^) (Figure 4D). Two enriched regions were identified when PGE was run with the 100 top-hits that appeared in both our MAT + MET15 screens. The larger region runs from 193592-264891bp and had five hits (*ICE2*, *AIM19*, *YIL060W*, *MMF1*, and *NEO1*) in a 49 ORF region. A smaller subsection was identified as the second hit, this region is 18,502bp long and contains 14 ORFs of which 3 were found in our top-hits (BP region 246389-264891) (p_adj_ = 2.72 × 10^−3^) (Figure 4D). A 13 ORF region of chromosome IX (BP region 83302-100501) contains two genes identified by at least two of the six compared studies (p_adj_ = 0.0299). One of the core CCT-complex genes, *CCT2*, is found in this region, and was identified by our study as well as Choy *et al.* Additionally, *CSM2* was identified by this study, and the primary screening conducted by Schmidlin *et al.* (Figure 4D).

#### Chromosome X:

No enriched regions containing more than 2 ORFs are found when running the 217 MAT locus genes alone. The MAT + MET15 100 gene analysis identifies three ORFs (*YJL009W*, *SYS1*, and *SPC1*) in a 23 ORF region (BP region 419849-458354) (p_adj_ = 9.01 × 10^−3^) (Figure 4E). A second enriched locus of interest is identified on chromosome X when the overlapping gene list from the multiple screen comparison is analyzed. This region is from 491074-517506bp and contains 3 top hits (*CPR7*, *PET191*, and *POL32*) in a 12 ORF region (p_adj_ = 4.18x10^−4^).

#### Chromosome XI:

Three ORFs (*YKR073C*, *ECM4*, and *YKR078W*) found in the MAT screen are identified in a 8 ORF region on chromosome XI (BP region 577829-586351) (p_adj_ = 7.51 × 10^−3^) (Figure 4F). This region is not found in our MAT + MET15 list. However, an overlapping region is found when the multiple screen comparison list is studied (BP region 584594-595940) (p_adj_ = 0.013) and contains 2 ORFs, YKR078W and NUP133, in a 5 ORF region.

#### Chromosome XV:

A large region is returned from PGE analysis of the MAT top-hits list (BP region 69376-139045) (p_adj_ = 2.88x10^−3^) with seven (*MED7*, *YOL134C*, *HRP1*, *PAP2*, *WSC3*, *NDJ1*, and *COQ3*) of the 46 ORFs in the region being found in that screen. A subsection of this region (BP region 69376-115808) (p_adj_ = 3.32 × 10^−3^) with four ORFs (*MED7*, *HRP1*, *PAP2*, and *WSC3*) out of a 33 ORF section is identified from the 100 gene MAT + MET15 list (Figure 4G). Analysis of the 91 overlap list from the multiple screen comparison did not return any enriched regions on this chromosome.

#### Chromosome XVI:

No enriched regions are identified from the 217 MAT locus gene list for chromosome XVI. However, when the 100 top-hits gene list from the MAT + MET15 screens is analyzed for chromosomal enrichment four ORFs in an 83 ORF region are found (BP region 600646-728613) (p_adj_ = 0.041). Two (*RPL36B* and *DIP5*) out of a 20 ORF region are found in a separate region of chromosome XVI (BP region 41043-76239) (p_adj_ = 0.046) and two other ORFs (*DDC1* and *YIG1*) out of a 9 ORF region (BP region 169769-181114) (p_adj_ = 0.019) are also identified when the list of 91 genes that were found in at least two separate screens are analyzed (Figure 4H). Within these regions several genes from heterozygous LOH/genome stability screens are found, *DIP5* was identified by both Strome *et al.* (2008) and Choy et.al., and *RPL36B* was identified by Choy *et al.* and this study.

### Fluctuation analysis of LOH candidate genes selected from data narrowing criteria

**Figure 5:**
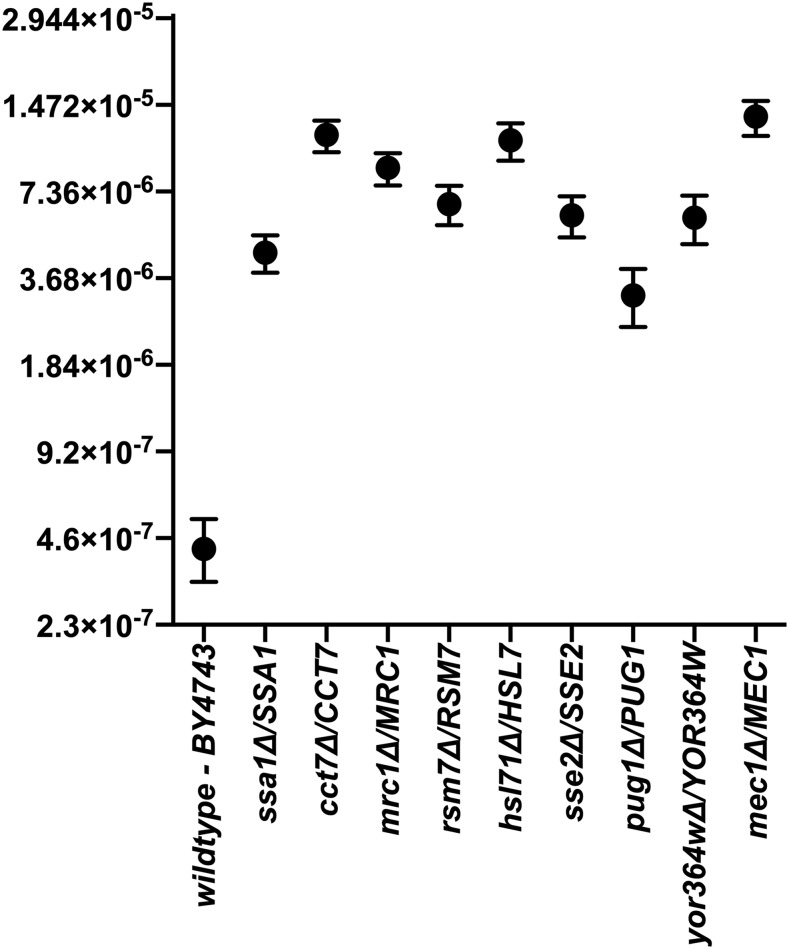
LOH rates at the *CAN1* locus due to nine separate heterozygous gene mutations. The data shown represents a combination of a minimum of two independent experiments. The black circle depicts the mean LOH rate with the tails showing the experimental 95% CIs. Non-overlapping 95% CIs, to the wildtype BY4743 strain, are considered significantly different as the 95% CI overlap method mimics a two-tailed, two-population *t*-test at the conventional *P* < 0.05 level with an improvement in type I error rate and statistical power when compared to a *t*-test, which has been found unsuitable for FA data analysis (Zheng 2015).

In an effort to determine the level of LOH induced by heterozygous gene mutations of interest we created strains capable of a quantitative fluctuation analysis assessment. Nine genes, *SSA1*, *CCT7*, *MRC1*, *RSM7*, *HS7*, *SSE2*, *PUG1*, *YOR364W*, and *MEC1*, were chosen for this assessment based on appearing within our screen and at least one secondary analyses discussed above (additional description of gene functions’ can be found in the discussion section below); the LOH rates of strains heterozygously mutated for these genes were then benchmarked using fluctuation analysis at the *CAN1* locus. *PUG1*, *YOR364W*, and *RSM7* were chosen because heterozygous mutations in these genes results in the highest LOH event scores in both our MAT and *MET15* assays. Futhermore *YOR364W* is categorized as a dubious open reading frame (ORF) indicating lack of clarity on if a protein/functional product is produced, and if so, an unknown function for that product. As such, *YOR364W* lacks characterization and falls in the *Unknown* categories for all three main GO terms: Molecular Function, Biological Process, and Cellular Component. *MRC1* was selected because it was indentified in three of the studies we analyzed, while *SSE2* was chosen because it was identified in two of the separate published studies (this study and Choy *et al.* that showed significant overlap) we analyzed for increasing LOH. To learn more about one of the clustered regions identified through PGE we selected three genes from the chromosomal II array, *MEC1*, *SSE2*, and *HSL7*. *CCT7* and *SSA1* were included in these analyses because they are part of the overrepresented gene ontology category of the CCT-complex, identified by both our study and Choy *et al.* Finally *CCT7*, *SSE2*, *MRC1*, *RSM7*, *HSL7* and *SSA1*, all have identified human homologs (see discussion) which could make their further investigation more relevant to LOH events in cancers. The SCDChet strains containing heterozygous mutations in *SSA1*, *CCT7*, *MRC1*, *RSM7*, *HSL7*, *SSE2*, *PUG1*, *YOR364W*, and *MEC1*, as well as the parental strain used to construct SCDChet – BY4743 – were transformed with a *can1*::*HIS3* cassette to render their *CAN1* locus heterozygous. Four independent fluctuation analysis experiments for the parental strain BY4743 were conducted to estimate the baseline LOH rate and 95% confidence intervals (see Figure 5) using the rSalvador package (Zheng 2002). The nine heterozygous gene deletion strains tested all showed significant increased LOH rates with 7- to 31-fold increases over the parental LOH rate (see Figure 5). These increased rates demonstrate that all of our secondary analyses were successful in narrowing candidates of interest whose single-gene deletion leads to a significant increase in LOH.

## Discussion

Through the work presented here we sought to identify heterozygous gene mutations with haploinsufficient impacts on LOH, as the homologs of these genes are particularly interesting targets for study since loss/mutation to only one allele could induce LOH-based cancer phenotypes. Multiple refinement strategies were employed to attempt to identify those genes with the greatest potential as candidate cancer susceptibility genes for futher study.

### Human homolog identification and cancer associations

Of the 217 genes identified as top-hits in the primary MAT locus LOH screening, we were able to identify 127 with a known human homolog (58.5%) utilizing YeastMine (https://yeastmine.yeastgenome.org) and NCBI Homologene (https://www.ncbi.nlm.nih.gov/homologene). Literature searches on these 127 genes identified 86 with a known association with cancer incidence (40% of the top-hits list, 68% of the “genes with human homologs” list) (see Table S8 for all top-hits with known human homologs). When the smaller list of mutants that increased LOH at both MAT + *MET15* was examined for human homologs in the same manner as above, 58 of the 100 genes (58%) were identified as having a human homolog, with 36 of the 58 human homologs with a known association to cancer (62%).

By again performing data comparisons to multiple independent studies, we are able to collect more information about genes with human homologs and those with known associations to cancers. Sixty-seven out of the 91 genes identified in at least two screens, have a known human homolog (74%). Including the genes identified in our screen (discussed above), a search of the current literature revealed that 54 of those 67 genes have an association with cancer (69%) (Table S9) (Sun *et al.* 2002; Leone *et al.* 2003; Mason *et al.* 2014; Abdel-Fatah *et al.* 2014; Hennecke *et al.* 2015; The *et al.* 2015; Wang *et al.* 2015; Taguchi *et al.* 2015; Dai *et al.* 2016). This serves as a positive control that performing these screens, and the multiple screen analyses, identifies genes, both with known human homologs that can be investigated for impacts on cancer development, as well as those that have already been linked to roles in cancer progression. The list of genes, with known human homologs not already associated with cancer phenotypes, are prime candidates as novel cancer susceptibility genes for future studies (as an example see discussion of *LSM4* below).

### Multiple screen comparisons for reproducibility of gene identification

In addition to conducting new screens to search for additional candidates, pooling the data from multiple, related screens, serves as a further layer to test reproducibility and enables identification of candidate genes that are likely contributors to genome instability.

*SSE2* encodes a heat shock protein 70 (HSP70) family member which functions as a nucleotide exchange factor for cytosolic Hsp70s during protein refolding (Easton *et al.* 2000; Shaner *et al.* 2005; Dragovic *et al.* 2006). This gene was identified in our primary MAT screen, reproduced in our secondary LOH screen at *MET15*, was also identified by Choy *et al.* as contributing to LOH at MAT in a haploinsuffient manner, and is found in an enriched region of chromosome II, and has a human homolog (*HSPH1)* with a known cancer association. The discovered relationship of heterozygous loss of this gene to induction of LOH events in yeast could provide an avenue for further mechanistics studies in this model system, a pathway for study design in mammalian cells, and a justification to screen for *HSPH1* alterations in additional cancer types.

Similarly, *LSM4* was identified in our MAT and *MET15* datasets (with one of the highest levels of LOH events observed in each), was also identified by Choy *et al.*, is found in an enriched region of chromosome V, and has a human homolog (*LSM4*), however there is no current direct cancer association. This gene encodes part of a protein complex involved in mRNA splicing, processing body assembly, and decay (He and Parker 2000; Beggs 2005). First characterized in systemic lupus erythemathosus patients, this protein is an intriguing prospect for further study, as SLE is a heterogeneous disorder, linked to increased incidence of many cancer types, however the data are inconsistent (Gayed *et al.* 2009; Song *et al.* 2018). Direct investigation into *LSM4* haploinsufficiency in a yeast model might allow investigation into the role of LOH events in disease progression.

Additionally, the seven genes found in three overlapping, related screens – *DCC1*, *SLX8*, *EMI2*, *MRC1*, *MCM21*, *RRM3*, and *ELG1* – qualify as strong candidates for further investigation due to consistently contributing to instability via LOH mechanisms under a variety of experimental conditions. Six of these genes are categorized in the *DNA Metabolic Processes* gene ontology and are further linked to chromosome organization (*DCC1*, *SLX8*, *MRC1*, *MCM21*, *RRM3*, and *ELG1)*. With known human homologs and cancer associations for all but *MCM21*, study of the mechanisms of association with increased LOH events could yield more information for inclusion in epidemiological studies as well as pathway information for targeted treatments.

Additional genes that induced the highest levels of LOH in our screens and showed statistically significant increases in LOH via fluctuation analysis are *RSM7*, *PUG1*, and *YOR364W*. *RSM7* encodes a small subunit mitochondrial ribosomal protein (Saveanu *et al.* 2001) and mutations in this gene were also picked up by Schmidlin *et al.* as increasing LOH events. Little else has been published about this gene and it is intriguing to consider how defects in a mitochondrial ribosomal protein induce nuclear genome instability. Further, while a human homolog has been identified, to date there has been no association with cancer making it a novel candidate cancer predisposition gene identified here. *PUG1* encodes a protoporphyrin uptake protein in the plasma membrane also involved in heme transport (Protchenko *et al.* 2008; Manente and Ghislain 2009). Choy *et al.* identified haploinsufficiency effects of loss of this gene on chromosome maintenance and Zhu *et al.* have shown increased colony sectoring in a CTF assay using the SCDChet (Zhu *et al.* 2015). Again, little additional information has been published on this gene. YOR364W, however, has had no direct work published about it, and is therefore another intriguing candidate. This ORF is still considered “dubious” and “unlikely to encode a functional protein” (https://www.yeastgenome.org/locus/S000005891). We however saw high levels of LOH induction in both our MAT locus and *MET15* locus screens as well as 14-fold increase in LOH at the *CAN1* locus via fluctuation analysis.

### Gene ontology enrichment

Further indication that our screen results are pertinent to the identification of human cancer-relevant gene mutations comes from our gene ontology analyses. On top of identifying many ontologies with clear cancer relevance such as *Chromosome Segregation*, *Mitotic Cell Cycle*, and *DNA Repair*, our GO analysis identified other ontologies which may hold relevance, but require more analysis of the members for candidate cancer susceptibility genes. For example, one of the top GO Term Finder ontology hits identified the *CCT-complex*; conserved from yeast to humans, some components of this complex have known cancer impacts. In yeast, systematic identical null mutations in each subunit of the eight core CCT complex proteins revealed varying phenotypic effects, indicating the possibility of secondary roles that individual subunits play in addition to cytoskeletal subunit folding as part of the CCT-complex (Amit *et al.* 2010). Secondary cellular roles of these subunits are additionally supported in research linking many, but not all, of the eight subunits to different cancer types. For instance, the homolog of one of our identified top-hits, *CCT2*, along with *TCP1*, has recently been linked as a driver mutation in breast tumor formation (Guest *et al.* 2015). As well, change in expression of *CCT8* has been linked to poor prognosis in glioma patients (Qiu *et al.* 2015). Studying the proteins of this complex could lead to more mechanistic insight on their secondary roles as well as determination of if other members of the complex play a role in cancer development. Furthermore, because members of the CCT-complex are essential genes, utilizing the SCDChet allowed for identification of this complex where it could not have been assessed using homozygous deletions; therefore, allowing for a more comprehensive look at genes contributing to genome instability through LOH events.

### Chromosome location enrichment clustering of genes involved in LOH

In the search for additional information that might aid in generating a better understanding of LOH we investigated the chromosome location of hits within our screen results alone, and further compared across multiple screens. These searches hold the potential to identify chromosomal regions and genes important in LOH events as well as to rule out genes that might have been identified due to artifacts in strain construction. On the one hand there are acknowledged errors, advantages, and disadvantages, for using a particular deletion collection (aneuploid strains, secondary mutations, incorrect genotyping) as well as screen-specific issues of strain reconstruction due to the nature of certain mutations (Hughes *et al.* 2000; Giaever *et al.* 2002; Deutschbauer *et al.* 2005; Yuen *et al.* 2007; Schmidlin *et al.* 2007; Andersen *et al.* 2008; Ben-Shitrit *et al.* 2012; Giaever and Nislow 2014). These known inconsistencies however do not negate the importance of the *S. cerevisiae* deletion collections as a tool for understanding genome instability, and the ability to apply knowledge to the study of higher eukaryotes. Conversely, enriched regions may contain regulatory sequences or identify the presence of a particularly important gene in the vicinity, and may point us to candidates with the most potential for further investigation. Because there is not conclusive evidence in all cases to confirm if a gene knockout is contributing to LOH due to regional effects, or via an independent mechanism, all possibilities need to be considered. We take the identified region of chromosome II as an example to discuss these models.

#### Chromosome II:

The large region of significance identified on the right arm of chromosome II, included seven MAT locus LOH screen identified genes within a 20 ORF region – *HSL7*, *CKS1*, *YBR137W*, *ATG42*, *BMT2*, *YBR144C*, and *APD1* and expanded to 19 identified hits in a region of 49 ORFs when run with the 91 gene multiple screen comparison top-hits list. Several possibilities exist for why this region was identified as an enriched locus for increasing LOH events.

The first possibility is that all/most of the genes in this neighborhood have independent impacts on LOH occurrences. While a few occasions of clustering of yeast genes with similar functions have been identified (Zhang and Smith 1998), it is possible that due to the large variety of gene functions that could be perturbed and lead to LOH that this region was not previously identified as one of these such clusters. Evolutionarily this clustering may have come into existance due to co-regulation of these genes for their involvement in genome stability maintenance.

A second possibility is that neighboring gene effects are driving LOH events through a single driver gene in the locus. For example, near the center of this region lies the gene *MEC1*. Orthologous to human ATR, *MEC1* is a critical mitotic checkpoint gene that plays an important role in responding to DNA damage as the cell navigates through the cell cycle (Harrison and Haber 2006; Bandhu *et al.* 2014). Further, mutations in *MEC1* have previously been shown to increase mitotic recombination events (Fasullo and Sun 2008) and decrease chromosome maintenance events (Choy *et al.* 2013), both of which could lead to increases in LOH; and has been identified in at least two previous screens looking for genes with impacts on genome stability (Stirling *et al.* 2011; Choy *et al.* 2013). *MEC1* was identified as a top-hit by Choy *et al.* indicating heterozygous loss of this locus can increase LOH events. As an essential gene this ORF would not be identifiable through screens utilizing the homozygous deletion collection. Other groups have reported neighborhood effects of KANMX insertions, however these are generally limited to adjacent genes within 600bp upstream or downstream of the driver locus. Ben-Shitrit *et al.* published a mechanism to predict such neighboring gene effects, however implementation requires a known protein-protein interaction network with anchoring proteins for the phenotype being measured (Ben-Shitrit *et al.* 2012). Since LOH events are caused by widely variable mechanisms via genes with widely variable functions we were unable to utilize their algorithm in this situation. Further study of this region to determine if all the identified ORFs are presenting as top-hits due to their proximity to a particular gene, like *MEC1*, or if they are contributing to genome instability through neighborhood-independent mechanisms could be accomplished through additional studies. This could take the form of individual knockout of every gene separately in a new strain, LOH rate quantification (such as via fluctuation analysis), and complementation assays, or through separate testing for expression levels of all of the genes in the region in each of the individual SCDChet strains representing the genes across this chromosomal region. Examining the extent of individual neighborhood effects in these clusters is a future direction that falls outside the scope of this study.

A third model is that the chromosomal region itself, potentially through TF binding, histone modification, replication firing, or three-dimensional architecture, may play roles in multiple loci being identified. Several studies have shown that a gene’s expression level tends to be similar to that of its neighbors on a chromosome (Zhang and Smith 1998; Kruglyak and Tang 2000; Cohen *et al.* 2000). If these are dosage sensitive genes, this might contribute to them having haploinsufficiency effects, which might account for the chromosomal region’s identification. To assess this possibility for the chromosome II region we searched for topologically associating domains (TAD) within a high-throughput chromosome conformation capture (Hi-C) dataset (Eser *et al.* 2017). A 130kb TAD was found to stretch across the region we identified (spanning from ∼450-580kb on Chr II), however the authors of that study report that the TAD-like domains they found were more strongly correlated with replication timing than with transcription.

A fourth model is that chromosomal regions are being identified as involved in increased LOH as a result of an artifact of how the knockouts were generated. To attempt to assess the possibility of region identification due to strain artifacts we have done two analyses. We started by running PGE with all 898 genes from the multiple screen comparisons (De Preter *et al.* 2008). The logic here is that if large regions are identified without individual genes being replicated this could tell us about the possibility for artifacts. Further, in running PGE in this manner we made note of which screen identified each gene, Table S10. We then further categorized each gene identified to the screen set-up and screens utilizing the same starting strains (*i.e.*, SCDChet *vs.* SCDChom *vs.* non-SCDC strains) were combined to determine if strain creation artifacts could be at play. We found that for the region in chromosome II, nearly all of the hits (16/19) were found from screens that utilized SCDChet, Figure S1. This could support the theory that something about these particular strains and the way they were made is leading to identification of this locus, but could also indicate a region high in genes with haploinsufficiency effects or essential genes not able to be identified by SCDChom screens (further substantiated by the fact that the other screen that identified two genes in this region did not use either deletion collection but completed insertional mutagenesis to assay for heterozygous effects (Strome *et al.* 2008)). We therefore moved to a second analysis to assess how strains were created as part of making the *S. cerevisiae* deletion collections. Since past artifacts have been identified when a set of strains were all created in the same lab (Lehner *et al.* 2007) we wanted to gauge if this chromosomal region of knockouts met this criteria. Information on strain creation from the Standford yeast deletion project website (http://www-sequence.stanford.edu/group/yeast_deletion_project/overlapping.html) identifies “lab 11” as having created this entire block of gene mutations, ending just where our identified region ends (at ORF YBR172C), however starting farther up (having created strains starting at YBR080C, our identified region starts at YBR127C). This unfortunately both supports and conflicts with the theory of artifact-induced LOH events in all strains made by this group. Supporting evidence is that this group did make all the gene knockout strains we identified. Conflictingly, however we would have expected to have identified all genes/most genes in the chromosomal region that encompasses all of the strains they created, not approximately half.

These models could apply to all of the PGE identified enriched regions and further investigation into all of these loci are warranted, but are outside of the scope of this mutant screen report.

### Essential genes

As less frequently evaluated loci, not assayed in haploid studies or studies of the homozygous deletion collection that are more frequently conducted, the cohort of essential genes from our screen are interesting targets for further study. 37 essential genes were identified in our primary screen *RPN6*, *LSM4*, *HSK3*, *PFS2*, *RLI1*, *SSY1*, *RSC3*, *BUR6*, *SLD3*, *ERG7*, *TTI1*, *HRP1*, *PSA1*, *FAP7*, *KRS1*, *FRQ1*, *MOB2*, *YJL009W*, *CCT7*, *SPC3*, *MED7*, *GPN2*, *RPO26*, *TSC13*, *MPS1*, *MET30*, *STH1*, *CCT2*, *CCT8*, *TUB1*, *MVD1*, *YOL134C*, *RKI1*, *RET1*, *MCM2*, *SMC1* and *NEO1*. Of these essential genes one, *CCT7*, was chosen for strain recreation to allow quantitative assesement of LOH rate due to heterozygous mutation. The significant 27-fold increase in LOH due to loss of this gene demonstrates its haploinsufficient impact on genome stability. Among this group, 32, have previously identified human homologs of which 23 have a known cancer association. Once again, analyses of these genes/proteins/pathways in a yeast system may help uncover additional mechanistic information important in understanding their roles in cancer development. Further, the nine genes (*LSM4*, *BUR6*, *ERG7*, *KRS1*, *SPC3*, *GPN2*, *TSC13*, *MUD1*, and *NEO1*) with known human homologs not already associated with cancer phenotypes are prime candidates as novel cancer susceptibility genes as they, like their yeast counterparts, may be able to induce haploinsufficient effects, not abiding the two-hit hypothesis, and therefore being more impactful gene mutations.

### Conclusions

Analyzing new screen data independently and then in conjunction with gene lists from relevant published works, as well as performing analyses beyond individual genes by looking at such items as ontology representation and positional gene enrichment, can increase the power of a study to identify genes of most importance. The studies a group might choose for comparison could be selected by literature search identification of relevant screens. We propose that this methodology of candidate narrowing allows the community to wade through the noise of data and focus on chromosomal deletions that play important roles in the phenotype or pathway of interest.
